# The voice of the plateau: a qualitative interview study of student management and educational practices in higher education in Xizang Autonomous Region

**DOI:** 10.3389/fpsyg.2026.1754922

**Published:** 2026-03-04

**Authors:** Fei Liu, Dejun Zhao, Xintang Li, Yang Yang

**Affiliations:** Department of Student Affairs, Xizang Agricultural and Animal Husbandry University, Linzhi, China

**Keywords:** labor education, experiential learning, identity, cross-cultural adaptation, internalization of responsibility, Xizang universities, qualitative research

## Abstract

**Background:**

In the multi-ethnic regions of China’s Xizang Plateau and its border areas, higher education not only shoulders the task of cultivating talent but also faces the dual challenges of promoting cultural understanding and ethnic unity. Labor education, as a crucial form of experiential learning, is considered a key mechanism for fostering students’ sense of responsibility, identity, and cultural adaptability. However, the underlying psycho-social-cultural mechanisms remain under-researched.

**Objective:**

This study aims to explore how labor education in Xizang universities promotes the internalization of responsibility, the construction of identity, and cross-cultural adaptation among students, while analyzing the mediating and guiding role of teachers between institutional governance and cultural inclusivity.

**Methods:**

Drawing upon theories of experiential learning, identity formation, and educational equity, this study employed a qualitative research approach. Semi-structured interviews were conducted with 34 participants (undergraduate, postgraduate, teachers, and experts) from universities in Xizang and surrounding plateau regions. The research followed Braun and Clarke’s six-phase thematic analysis method, identifying core themes and mechanisms through open, axial, and selective coding.

**Results:**

The analysis yielded four key findings: (1) Students exhibited emotional resistance to excessive management while rationally acknowledging institutional order; (2) Labor education facilitated the psychological internalization of responsibility and the formation of cultural belonging through embodied experiences; (3) The role of teachers shifted from “managers” to “cultural mediators” and “growth guides,” bridging understanding between tradition and modernity; (4) A structural disconnect exists between national policies and local practices, necessitating the establishment of flexible, feedback-oriented educational governance mechanisms.

**Conclusion:**

This study reconceptualizes labor education as a socio-educational governance mechanism that fosters identity integration, social cohesion, and emotional resilience. It reveals the psychological pathways through which experiential learning transforms individual and collective identities within multicultural educational contexts. The research offers novel theoretical perspectives for the fields of cross-cultural psychology and educational psychology, along with practical implications for educational reform in multi-ethnic regions.

## 1 Introduction

Globally, higher education systems are undergoing profound transformations driven by inclusivity, equity, and cultural sustainability (Nguyen et al., 2024). Universities are not merely sites of knowledge production but also social spaces where diverse identities, values, and worldviews converge, collide, and are reshaped (Koshy et al., 2022). There has been extensive discussion in international academia regarding student management and educational governance within mainstream contexts (Münch and Wieczorek, 2022). In recent years, the role of labor education in China’s higher education system has received considerable attention, emphasizing the importance of leveraging the strengths of professional labor education, adhering to the principle of “fostering virtue through education”, and implementing collaborative education (Yang, 2023). The nation is committed to cultivating talent with comprehensive moral, intellectual, physical, esthetic, and labor development (Luo, 2023). Labor education is currently a focal point of educational efforts, enabling university students to enhance their labor spirit, value orientation toward labor, and skill levels (Xiangjiao et al., 2021). In this context, labor education transcends the mere cultivation of practical skills and responsibility; it serves as a crucial mechanism for shaping civic values and social adaptability (Zhang and Ye, 2024). In the Xizang Plateau, border regions, and multi-ethnic highland areas of China, its significance is even more profound. Labor education is deeply rooted in a multicultural (Bakhtiyorovich, 2020) and diverse social environment (Ren and Xie, 2023), closely intertwined with issues of identity, cultural integration, and social cohesion.

Despite the growing emphasis on labor education in China (Yan, 2021), it has garnered scant attention in international academia. Existing research predominantly concentrates on related concepts such as service-learning (Chan et al., 2020) and experiential learning (Kovaric and Gingell, 2024) in higher education, with a strong focus on Western educational contexts. Currently, case studies examining labor education within unique socio-cultural environments, particularly in plateau and border regions like Xizang, China, are exceedingly rare. Furthermore, existing studies tend to prioritize policy research (Ming, 2023) and institutional analysis (Huang, 2023), with insufficient focus on the lived experiences of students and teachers (Dawes et al., 2021). Consequently, a thorough exploration of the mechanisms through which labor education cultivates responsibility, shapes identity, and promotes cultural adaptation within higher education environments (Antoniadou and Quinlan, 2021) remains underexplored.

This study aims to address research gaps in this field by conducting in-depth qualitative interviews with students, teachers, experts, and other stakeholders in Xizang, border regions, and multi-ethnic plateau areas of China. Labor education research is poised to undergo a significant historical transformation (Xiong, 2023), representing a theoretical articulation of labor education and its value choices. All sectors of society should establish correct value concepts of labor and enhance students’ practical abilities in labor education (Xu, 2022). Specifically, this research investigates the practices of labor education in universities within the unique geographical context of Xizang, China, examining how it shapes students’ sense of responsibility, identity, and cultural adaptation, as well as the role educators play in integrating governance and values through labor education. By deeply exploring the authentic voices of students and teachers, this study not only documents the practices of labor education within the distinct cultural backdrop of plateau regions but also analyzes its broader educational and practical implications. Based on the foregoing considerations, this study sets out to explore the following three core research questions (RQ):

*RQ1*: What are the specific practical pathways and localized characteristics of labor education in Xizang universities?

*RQ2*: How does labor education influence students' formation of responsibility, construction of identity, and process of cultural adaptation?

*RQ3*: What role do teachers play in labor education, and how can they serve as cultural mediators and governance collaborators?

**Figure 1:**
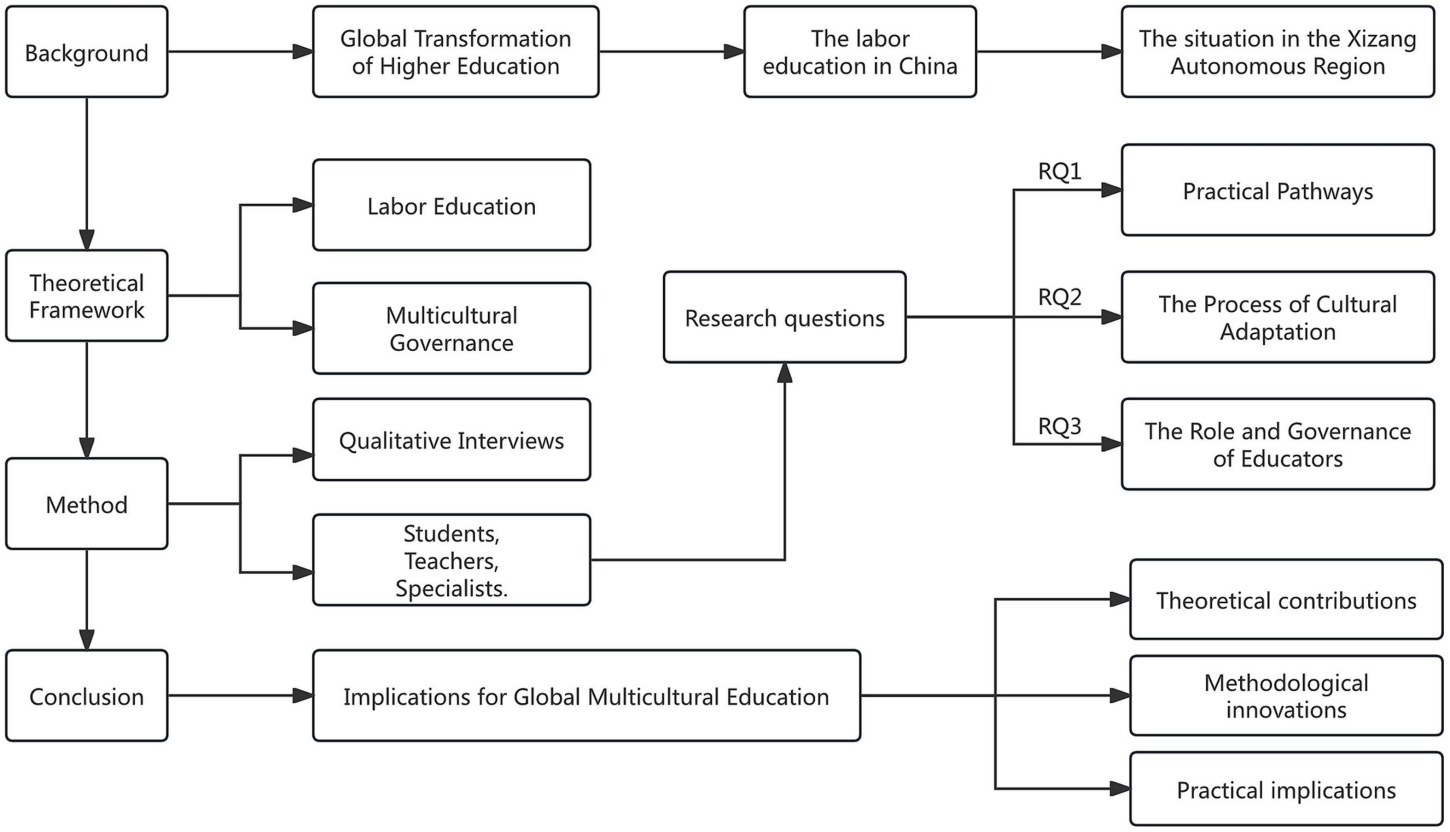
Theoretical framework.

Through qualitative interview research, this study deeply investigates the dynamics of labor education in Xizang higher education and contributes to comparative studies of multicultural and experiential education. By contextualizing labor education within the unique socio-cultural environment of Xizang universities, this research expands the international discourse on experiential learning and service-learning, grounded in the theoretical framework presented in Figure 1. In China, the practice of labor education transcends a mere pedagogical tool, functioning instead as a governance mechanism that intertwines individual development with collective identity and social integration.

This study successfully theorizes labor education in universities within China’s Xizang Autonomous Region, transforming it from a regional administrative practice into a universally applicable analytical framework for multicultural educational governance. Three innovative contributions are presented: (1) Theoretical Innovation. A novel theoretical perspective of “Labor Education as a Nexus for Multicultural Governance” has been constructed. (2) Practical Innovation. The micro-level realization pathway from “inclusion to integration” has been elucidated. (3) Methodological Innovation. A bottom-up research path for cross-cultural education has been established.

This research not only deepens the understanding of the distinctiveness of labor education in universities on the Xizang Plateau and in border regions but also offers new theoretical tools and practical references for global multicultural educational governance. Particularly within the context of globalization, where interactions and integration among diverse cultural groups are increasingly frequent, achieving deeper social cohesion through education has become a critical issue. It also demonstrates the unique role of labor education in fostering cultural understanding, enhancing social responsibility, and constructing identity, providing a transferable model for policymakers in other multi-ethnic, multicultural regions and advancing academic recognition of the complexity of educational practices.

## 2 Literature review and theoretical framework

### 2.1 Theoretical evolution of service-learning to labor education and its limitations

The international academic community has a long-standing tradition of discussing student management and experiential education in higher education. Originating from Dewey’s concept of “learning by doing,” experiential learning theory emphasizes the internalization of knowledge and cognitive development through direct experience (Marougkas et al., 2023). Service-learning, which evolved from this foundation, integrates community service with academic curricula (Wei, 2023) and is widely regarded as an effective pathway for cultivating students’ civic responsibility, social skills, and critical thinking. Furthermore, the theory of communities of practice (Goodchild, 2014) highlights that learning is a process of social participation, wherein individuals construct identity and a sense of belonging within shared practical activities.

While these theories provide a crucial cornerstone for understanding the practical components of education, their research contexts present discernible limitations. Firstly, existing theoretical paradigms have largely emerged and developed within Western individualistic cultural frameworks, prioritizing the cultivation of individual potential and critical awareness. Consequently, they offer insufficient exploration of education’s structural role in shaping national identity and promoting cultural integration. Secondly, although “service-learning” and “labor education” share formal similarities, labor education within international academic discourse is often narrowly defined as “vocational skills” (Schulz et al., 2023) training or livelihood education. Its multifaceted function as a comprehensive educational vehicle, particularly in linking cultural heritage, identity construction, and social governance, has not received adequate attention or theoretical elaboration. This theoretical myopia limits its explanatory power for educational practices in specific regions of non-Western, multi-ethnic nations.

Despite the widespread application of experiential learning and service-learning theories in educational psychology, comprehensive psychological research still lacks a systematic exploration of the mechanisms underlying the internalization of responsibility and identity construction in “labor education.” This is particularly true regarding studies on individual psychological transformation processes in cross-cultural and multi-ethnic educational contexts. This study explicitly contrasts the theoretical differences between service learning, experiential learning, and the present research.

### 2.2 Research trajectories of labor education in a global context and specific concerns of Xizang higher education

Within the macrocosm of global educational research, organizations like UNESCO advocate for education to foster global citizenship, sustainable development, and intercultural understanding (Akçay et al., 2024). Against this backdrop, experiential learning theory, originating from Dewey’s “learning by doing” philosophy, and its subsequent development into the service-learning model, have been widely recognized globally as effective pathways for cultivating students’ social responsibility (Ruan, 2025), critical thinking, and community engagement skills. Service learning emphasizes the integration of academic study with organized community service (Álvarez-Vanegas et al., 2024), aiming to achieve the dual objectives of societal contribution and learner development (McKinnon et al., 2017). This necessitates a paradigm shift not only in traditional educational philosophies and models but also, through a process of value clarification and situational analysis, the cultivation of innovative spirit within students’ burgeoning sense of responsibility (Qi, 2023). Concurrently, theoretical discussions on “communities of practice” posit that learning is inherently a social participation, wherein individuals construct knowledge and identity through shared practical activities.

However, mainstream global academic discourse is rooted in the Western individualistic tradition, with a primary focus on stimulating personal potential and critical consciousness (Shin and Rubio, 2022). This discourse exhibits a deficiency in exploring the structural functions of education in multi-ethnic nations, such as the construction of national identity and cultural integration. Labor education in international literature is often narrowly defined as “vocational skills training” (Iliescu et al., 2025), failing to fully theorize its comprehensive educational function in linking cultural inheritance, identity formation, and social governance. This theoretical limitation renders existing research less explanatory when interpreting educational practices in plateau frontier regions with unique geographical, cultural, and political contexts, such as Xizang in China.

The academic concerns within Xizang higher education exhibit a distinct duality, reflecting a profound understanding of management practices and the cultural adaptability of educational content, emphasizing the imperative to respect the cultural psychology and local knowledge systems of multi-ethnic students. Research in Xizang universities is also deeply embedded within its specific national context, carrying the unique mission of integrating the concept of “Forging a Strong Sense of Community for the Chinese Nation” (Zhang, 2023) throughout the entire educational process. This reflects the universal phenomenon of education serving social integration goals within particular political and cultural contexts. Despite global theoretical discussions and localized policy concerns, significant research gaps remain. Specifically, empirical studies within multi-ethnic educational settings are lacking in the domains of labor education and educational psychology, which are crucial for elucidating the dynamic processes through which labor education, via its mechanisms, promotes the internalization of responsibility, identity formation, and cultural adaptation.

Compared to the internationally accepted theories of “service learning” and “experiential learning,” this study focuses on: (1) Situating labor education within a multi-ethnic, multicultural governance context, revealing its function as a “socio-educational governance mechanism” rather than merely focusing on individual development; (2) It empirically uncovers the psychological mechanisms through which labor education fosters responsibility internalization, identity formation, and cultural adaptation, filling a gap in educational psychology regarding psychological transformation processes within multi-ethnic educational settings; (3) It proposes a progressive model of “from action to identity,” offering a new theoretical perspective for understanding labor education’s role in promoting social cohesion and community consciousness construction.

### 2.3 The deficient integration of interculturalism, identity, and educational equity

Current research on student management and labor education in Xizang universities largely remains at the institutional and policy explication level, lacking analytical frameworks that can systematically dissect individual psychological experiences and their interplay with social structures. A significant disconnect exists between mainstream international educational theories and the contextual realities of Xizang practice. Predominant theoretical paradigms in experiential learning, service learning, and related fields, largely originating from Western individualistic cultural backgrounds (Thobani, 2018), primarily focus on cultivating students’ critical thinking (van Peppen et al., 2021), personal potential, and social engagement capabilities (Corazza and Glăveanu, 2020), and mass schooling facilitated nation-building within the modernization process (Tao, 2023). However, when these theories are transplanted into the unique milieu of Xizang—a region characterized by its high-altitude geography, frontier location, multi-ethnic culture, and a special mission of political integration—their explanatory power becomes constrained. They struggle to fully account for how labor education in this context transcends individual development to effectively serve macro-structural functions such as cultural integration, the construction of national identity, and even the cohesion of frontier societies. This limitation in theoretical perspective creates blind spots in the international academic community’s understanding of educational practices in non-Western, multi-ethnic regions and prevents the distinctive educational explorations within Xizang universities from being adequately integrated into global educational discourse.

A discernible rupture exists between macro-level policy directives and micro-level individual experiences. Existing studies predominantly adopt a top-down perspective, concentrating on textual interpretations of national educational policies, particularly the requirements for labor education within the “cultivating virtue and nurturing talent” and “five-pronged education” (Yuan, 2023) initiatives. However, these studies often fall short in revealing the complex interactions that occur when these macro-level policies are implemented at the grassroots level in Xizang universities, particularly in relation to specific campus conditions, faculty, and student demographics. How are uniform policy requirements executed, adapted, or even circumvented in universities with varying resource endowments? How do students and faculty, as the ultimate recipients of these policies, understand, perceive, and respond to them? How is their agency manifested within the interstices of the system (Tu and Sun, 2025)? These dynamic, vibrant, and tension-filled processes of “policy practice” are not adequately represented in current research.

A decoupling is evident between the description of educational practices and the in-depth exploration of theoretical underpinnings. While numerous studies document the forms and content of labor education in Xizang universities, most remain at the level of activity description and experience summarization (Cheng, 2023), failing to engage in systematic, in-depth dialog between rich practical experiences and core sociological and educational theories such as identity formation, educational equity, cultural capital, and multicultural education. For instance, how does labor education serve as a field for students, particularly minority students, to negotiate their cultural identities, regional affiliations, and their sense of belonging to the Chinese nation? Do current management practices and educational resource allocations serve to dismantle or reproduce certain structural inequalities? Existing research lacks robust analytical tools and a conscious effort toward theoretical construction to address these deeper theoretical questions. Furthermore, current research has not, from a psychological or educational psychological perspective, elucidated how labor education in multi-ethnic university settings fosters a sense of responsibility, cultural identity, and belonging through emotional resonance and social interaction, nor has it provided a systematic analysis of its psychological construction mechanisms.

### 2.4 An integrated analytical framework for research theory

This study introduces three interconnected theoretical threads that collectively form the framework for subsequent data analysis. (1) Grounded in the theory of experiential education, and focusing on the processes of embodied learning, knowledge construction, and value formation within labor education, this research examines labor education as a practical activity, emphasizing its inherent processes and experiences. (2) Identity and belonging theories are crucial for deepening our understanding. They direct this study to explore: Within the multi-ethnic plateau environment, how does labor education become a space for students to engage in individual identity exploration and cultural negotiation? (Du and Wang, 2022). How does it influence students’ sense of identity and belonging to themselves, regional cultures, and even the Chinese nation? (Kandiko Howson and Lall, 2022). (3) Theories of educational equity and cultural diversity provide value orientation and critical perspectives. Do current management and educational practices replicate structural inequalities, or do they genuinely promote cultural inclusion and equitable participation? This helps this study move beyond superficial cultural adaptation to a deeper concern for cultural symbiosis and equitable empowerment.

**Figure 2:**
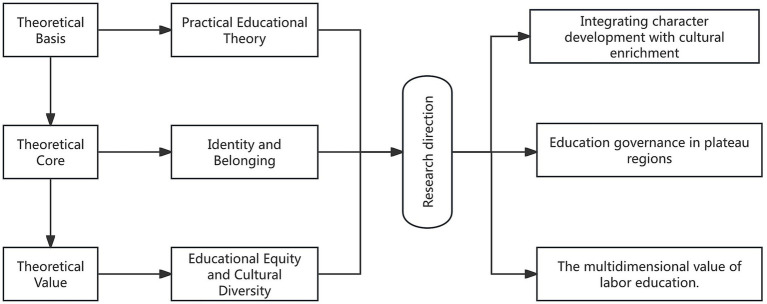
Theoretical framework of the research.

Through the intertwining of these three theoretical threads, this study reconceptualizes labor education in China’s Xizang Plateau, border, and multi-ethnic regions as a multidimensional governance and integration mechanism—encompassing pedagogical practice, cultural practice, and governance practice that promotes social cohesion. This framework not only powerfully addresses the aforementioned theoretical gaps but also ensures that the research findings can engage in broader international academic discourse, facilitating a leap from regional case studies to theoretical insights (Figure 2).

## 3 Methodology

### 3.1 Research design

This study employed a qualitative research approach, consistent with social science methodologies that focus on identifying and interrelating recurring themes within individual perceptions as revealed through language and communication (Yang, 2024). Utilizing purposive maximum variation sampling (Liu et al., 2025), we selected participants for semi-structured interviews. These interviews aimed to gather diverse perspectives from undergraduate and graduate students, faculty, and experts in the field within the plateau region. The objective was to explore their views, experiences, and practical strategies concerning the design, implementation, and challenges of labor education in higher education institutions on the plateau. Through this interview design, we sought to comprehensively elicit current student perceptions and cognitions of labor education, faculty practices and challenges, policy formulation and execution, and the profound experiences of university students across different age groups regarding labor education in the plateau region. The study intends to delve into a deep understanding of the actual conditions, challenges, and proposed countermeasures for labor education in higher education institutions within China’s Xizang Autonomous Region, as perceived by undergraduate and graduate students, university faculty, and subject matter experts.

### 3.2 Informed consent and ethical approval

Prior to the commencement of interview data collection for this study, securing informed consent from all participants and obtaining relevant ethical approval were critical steps. This research strictly adhered to the ethical norms of academic inquiry, ensuring the legality and ethical integrity of the research process. Before inviting participants to engage in interviews, the research team provided each potential respondent with a detailed explanation of the study’s objectives, methodologies, anticipated outcomes, and potential risks. This explanation ensured that participants could make a voluntary decision to participate based on a full understanding of the research content. Concurrently, the research team informed participants of their right to withdraw from the study at any time without incurring any adverse consequences. Upon receiving explicit consent from participants, the research team executed written informed consent forms with each individual. These forms documented the participants’ understanding of the research content and their agreement to participate, alongside detailed assurances from the research team regarding the protection of participants’ privacy and data. The signing of these consent forms established a clear legal framework between the research team and the participants, providing legal assurance for the smooth progression of the study.

Furthermore, this study rigorously complied with the requirements of the Declaration of Helsinki (Association, 2013). Prior to the formal interviews, we obtained official approval from the Institutional Review Board of Xizang Agricultural and Animal Husbandry University and accordingly, an ethics review approval letter (Approval No.: 2025-XZNM-0058) was issued. The Ethics Review Board conducted a comprehensive review of the research protocol, encompassing the reasonableness of the research objectives, the scientific rigor of the research methodology, and the measures for participant protection, among other aspects. Only after receiving approval from the Ethics Review Board did the research team formally commence the collection of interview data. This step ensures the ethical compliance of the study and provides a significant safeguard for the reliability and validity of the research findings.

### 3.3 Interview participants

The participants for this study were recruited using purposive maximum variation sampling. The interviews were conducted between August 28 and September 26, 2025. A total of 34 participants from diverse backgrounds were invited, including: three undergraduate students from different disciplines (Business Administration, Law, and Computer Science and Technology) in their 2023–2025 academic years at University A located in the Xizang Plateau, border, and multi-ethnic regions. Additionally, two postgraduate students in Applied Economics (Class of 2025) and two experts specializing in research on the “sense of community of the Chinese nation” were included. From University B we recruited one undergraduate student in Management (Class of 2023), one undergraduate student in Accounting (Class of 2024), two postgraduate students in Education (Class of 2024), two faculty members from the medical school, and relevant administrative leaders. University C contributed five undergraduate students from various majors (Ecology, Energy and Power Engineering, Animal Science, Tourism Management, and Plant Protection) in their 2022–2025 academic years. Furthermore, two postgraduate students in Preventive Veterinary Medicine (Class of 2022) and three students in Resource Utilization and Plant Protection (Class of 2024) participated. The group also included two full-time labor education instructors, the director of the Ideological and Political Education Research Office, and three frontline counselors. These participants represented a wide array of academic disciplines, professional fields, and work environments, ensuring the breadth and diversity of the research findings. All participants voluntarily engaged in this study. Prior to the interviews, participants were thoroughly briefed on the research objectives and potential implications (Rodrigues and Verstraten, 2021). This study employed purposive maximum variation sampling, continuously collecting data until no new core themes emerged from subsequent interviews. During interviews, each segment was analyzed immediately and compared with existing data. After reaching eight teacher participants, the subsequent three interviews only enriched existing themes without introducing new ones, prompting the cessation of teacher data collection. Student data collection involved 10 undergraduate and 9 graduate student interviews. Significant differences in experiences were found across grade levels and majors, but no new core themes emerged, leading to the termination of student data collection. Expert and teacher sample sizes were determined based on representativeness within their respective fields. Overall, the sample of 34 participants adequately captured diverse perspectives from Tibet’s plateau, border regions, and multi-ethnic areas, ensuring research depth and diversity. During the data collection phase, essential information such as age, gender, academic background, and years of work experience was recorded and subsequently anonymized for analytical purposes.

### 3.4 Theoretical framework and interview question design

This study systematically designed interview questions based on an integrated theoretical framework developed through a literature review, ensuring a close alignment between data collection and theoretical dimensions. Practice education theory served as the foundational perspective (Jiménez and Ramírez, 2024), emphasizing the embodied learning characteristics of labor. Consequently, the interview questions focused on examining the practical processes and student experiences within labor education. For instance, participants were asked to describe memorable labor experiences and their associated feelings to elucidate the unified shaping mechanism of knowledge, skills, and values. Identity and belonging theories provided a deepening perspective, focusing on the identity construction functions of labor education. The corresponding questions explored how participation in labor influences students’ perceptions and emotional changes regarding national identity, regional belonging, and the sense of community of the Chinese nation, thereby revealing its role as a field for identity negotiation. Theories of educational equity and cultural diversity offered a critical lens (Foster et al., 2023), guiding attention to issues of fairness and inclusivity in practice. Related questions investigated differences in participation opportunities and resource acquisition among students from various backgrounds in labor education, as well as the extent to which the curriculum content reflects cultural diversity, scrutinizing structural equity issues. The three theoretical frameworks guiding this study informed the design of interview questions, coding decisions, and interpretations. Specifically: (1) The theory of practice-based education directed our focus on the embodied nature of labor learning, prompting coding to emphasize details of labor experiences, emotional responses, and cognitive shifts; (2) Identity and belonging theory guided analysis of labor education’s impact on students’ perceptions and emotions regarding ethnic identity, regional belonging, and awareness of the Chinese national community, with coding focusing on themes like identity negotiation and cultural belonging; (3) Educational equity and cultural diversity theory aided examination of fairness and inclusion in practice, with coding emphasizing themes like disparities in resource access and cultural expression. Through systematic theoretical guidance, we avoided parallel presentation of frameworks, instead integrating them organically into the analytical process. By operationalizing these three theoretical threads into specific interview question dimensions, this study achieved a systematic integration of the theoretical framework and empirical data, laying a solid foundation for an in-depth analysis of the complex interplay of labor education across practice, identity, and equity dimensions.

### 3.5 Interview data collection

The semi-structured interview guide for this study was reviewed by five domain experts and finalized after three pilot interviews (Appendix 1). Each interview lasted approximately 45 min. The interview protocols for each participant group were meticulously designed to encompass inquiries across multiple dimensions, including student management and personal experiences, academic development and educational practices, educational administration and faculty roles, and institutional-level and educational development aspects. To ensure the comprehensiveness and accuracy of the data, all interviews were audio-recorded. Following the interviews, the recordings were transcribed verbatim to capture every detail of the dialog, facilitating subsequent in-depth analysis.

### 3.6 Data analysis

In this study, the data analysis process adhered to the theoretical framework and methodology of qualitative analysis proposed by Braun and Clarke’s (2006) and Craver (2014), and Ralf Buckley’s 10-step qualitative research analysis method. A three-level coding process was employed to progressively refine thematic issues pertinent to student management and educational practices (Skjott Linneberg and Korsgaard, 2019). To ensure coding consistency, this study employed a three-stage coding process, establishing a rigorous inter-coder reliability mechanism. Initially, five researchers independently conducted open coding to generate preliminary coding tables. Subsequently, the research team convened coding meetings to discuss any discrepancies, reaching consensus through collective deliberation and referencing the theoretical framework. For codes where agreement could not be reached, an iterative “code-discuss-recode” process was implemented until all coders achieved over 90% agreement. Finally, a highly experienced coder, the principal investigator, performed the final review. Throughout the coding process, a total of 16 divergences were identified; 85% were resolved through group discussion, and the remaining 15% were settled by consulting the theoretical framework. This mechanism ensures the rigor of the coding process and the reliability of the findings.

### 3.7 Data saturation and data credibility

During the data collection phase of the interviews, to rigorously ensure the credibility of this research, we implemented a multi-faceted approach: (1) Employing triangulation to enhance data reliability by collecting diverse sources from distinct participant groups, namely students, educators, and domain experts; (2) Validating emergent findings through member checking, where preliminary analytical outcomes were shared with select participants to ascertain their accuracy; (3) Mitigating researcher bias by maintaining a reflective journal to document subjective judgments and potential influences throughout the analytical process; (4) Fostering analytical objectivity and consistency via regular discussions within the research team. Collectively, these methodological safeguards underpin the trustworthiness, dependability, and confirmability of our study. We continued conducting interviews until theoretical data saturation was achieved. Our predefined criterion for data saturation was established as the point where newly acquired interview data on labor education no longer yielded new core themes or concepts, and the supplementation to existing themes and concepts was minimal (Braun and Clarke, 2019). In practice, as the interviews progressed, we analyzed the content of each interview in real-time and compared the localized and culturally inherited themes and concepts of labor education from new interview data with those already collected. For instance, in interviews with university faculty, after interviewing eight participants, we observed that the subsequent three consecutive interviews with teachers yielded information that primarily enriched existing themes rather than introducing new core themes. These themes focused on “faculty role positioning, cultural mediation,” and “student engagement, identity construction,” but no novel core themes emerged. Following discussion and evaluation by the research team, we determined that data saturation regarding faculty positioning had been achieved, and thus ceased data collection for faculty roles and positioning. This approach ensured the comprehensive acquisition of necessary and valid information while preventing unnecessary resource expenditure.

### 3.8 Interviewer reflection

To ensure the effective implementation of interviews, we recognized the critical importance of interviewer reflection. Consequently, the interviews in this study were conducted by Fei Liu and Xintang Li both of whom possess research, learning, and practical experience in university student management and labor education, and have undergone professional training in interviewing techniques. Throughout the interview process, interviewers maintained a neutral and objective stance. Concurrently, they actively reflected on the influence of their own roles and behaviors on the interviews (Gesch-Karamanlidis, 2015). During the interviews, particularly when addressing sensitive questions, interviewers meticulously considered the appropriateness of their questioning methods and the potential for eliciting specific responses from interviewees. Subsequently, they would adjust their interview strategies accordingly to ensure the collection of authentic and objective information (Elliott, 2018). This study’s investigators are researchers in the field of student management and labor education within higher education institutions. Their insider perspective presents both an advantage and a challenge. While our intimate understanding of the Tibetan higher education environment and management culture offers valuable context, it also carries the risk of influencing data interpretation. We acknowledge the inherent power dynamic between researchers and participants, where we represent academia and participants represent institutional management. To mitigate this, we have implemented several strategies: (1) Prior to interviews, participants were fully informed about the study’s objectives and our role as academic researchers, explicitly stating that the interviews were not evaluative. (2) We maintained neutrality throughout the interview process, actively avoiding leading questions. (3) Reflexive journaling was employed to document observed power dynamics during interactions. (4) External experts familiar with Tibetan culture were engaged to participate in the analysis, providing an objective viewpoint. We specifically considered how to approach the sensitive topic of administrative rigidity, aiming to prevent participants from feeling scrutinized or accused, thereby fostering genuine expression of their experiences.

## 4 Results

### 4.1 Demographic information of participants

This study obtained rich primary data through in-depth interviews with 10 undergraduate students, 9 graduate students, 8 university faculty members, and 7 experts. Among the 34 participants, 21 were male and 13 were female, a gender ratio that to some extent reflects the current gender structure in some subject areas within Xizang higher education institutions in China (Table 1). In terms of age distribution, 13 participants were between 18 and 28 years old, primarily undergraduates and junior graduate students; 14 participants were between 29 and 39 years old, mainly young faculty, counselors, and some doctoral students; 4 participants were between 40 and 49 years old, mostly senior faculty or middle managers; and 3 participants were between 50 and 59 years old, predominantly domain experts and senior administrators (Table 2). The uniform distribution of participants in terms of identity, educational background, age, and gender effectively ensured the diversity of the research sample, laying a solid foundation for understanding the complexity of student management and labor education in Xizang from multiple perspectives, thereby enhancing the reliability of the research findings.

**Table 1:** Basic characteristics of research participants.

Type 1	Type 2	Number of participants	Percentage (%)
Identity	Undergraduate	10	29.41%
	Graduate student	9	26.47%
	University Teacher	8	23.53%
	Expert	7	20.59%
Educational Background	Bachelor’s degree	13	38.24%
	Master’s degree	16	47.06%
	Doctorate	5	14.71%
Gender	Male	21	61.76%
	Female	13	38.24%
Age	18–28	13	38.24%
	29–39	14	41.18%
	40–49	4	11.76%
	50–59	3	8.82%

**Table 2:** Interview participant identification codes (IDs) and corresponding citations.

Identity	Code	Gender	Educational background	Citation in the main text
Undergraduate	B1	Female	Bachelor’s degree	/
	B2	Male	Bachelor’s degree	Participated in the “Highland Greenhouse” project, sharing personal experiences.
	B3	Male	Bachelor’s degree	Law major, discussing institutional necessity and procedural fairness.
	B4	Male	Bachelor’s degree	/
	B5	Male	Bachelor’s degree	From the mainland, understanding management systems as “safety guardrails.”
	B6	Female	Bachelor’s degree	An undergraduate student shared their adverse experiences resulting from excessive management.
	B7	Male	Bachelor’s degree	Senior student, discussing trust and self-management.
	B8	Male	Bachelor’s degree	From the mainland, participated in traditional festival labor, discussing cultural adaptation and regional integration.
	B9	Male	Bachelor’s degree	/
	B10	Male	Bachelor’s degree	/
Postgraduate	Y1	Female	Master’s degree	Tibetan students engaging in traditional handicraft labor, discussing cultural identity.
	Y2	Female	Master’s degree	Participating in pastoral area technology services, discussing professional responsibility.
	Y3	Male	Master’s degree	Describing the collaborative relationship with mentors.
	Y4	Male	Master’s degree	/
	Y5	Female	Master’s degree	Describing the collaborative relationship with mentors.
	Y6	Male	Master’s degree	/
	Y7	Male	Master’s degree	/
	Y8	Male	Master’s degree	/
	Y9	Female	Master’s degree	/
Teachers	T1	Female	Bachelor’s degree	Frontline educators discuss the localization and adaptation of labor education content.
	T2	Male	Bachelor’s degree	A Teacher’s Perspective on Implementing Labor Education.
	T3	Male	Bachelor’s degree	Student management cadres discuss the humanized fine-tuning of dormitory management.
	T4	Male	Master’s degree	Mentors discuss the evolution of the mentor’s role.
	T5	Male	Master’s degree	Counselors discuss the transformation of management philosophy and democratic participation.
	T6	Female	Master’s degree	Teachers discuss humanistic care and support beyond academics.
	T7	Male	Bachelor’s degree	A seasoned educator with 15 years of experience working in Tibet discusses the role of a “cultural bridge.”
	T8	Female	Master’s degree	/
Experts	P1	Male	Master’s degree	/
	P2	Male	Master’s degree	Xizang Plateau experts discuss labor education as a crucible for community consciousness.
	P3	Female	Doctorate	Leaders
	P4	Female	Doctorate, Prof.	Leaders call for the establishment of a two-way feedback mechanism.
	P5	Female	Master’s degree	Experts discuss resource bottlenecks as constraints.
	P6	Female	Master’s degree	They emphasize insufficient localized adaptive capacity.
	P7	Female	Doctorate, Prof.	A deficiency in policy feedback mechanisms.

### 4.2 Data analysis and theme extraction

This study transcribed over 15 h of interview recordings verbatim, resulting in approximately 60,000 words of textual data. Data analysis strictly followed Braun and Clarke’s (2006) six-phase thematic analysis method, augmented by a three-level coding process.

(1) In the initial phase, the research team extracted 40 initial codes (e.g., “institutional necessity,” “positive experiences with labor education,” “challenges of cultural differences,” “insufficient resources”) from the text through open coding. Subsequently, axial coding categorized these codes into several broad categories, such as “student experiences,” “teacher roles,” “management systems,” and “cultural dimensions.”
(2) Through axial coding, researchers systematically grouped these seemingly disparate but intrinsically linked initial codes under several more generalized core categories, including “student experiences,” “teacher roles,” “management systems,” “cultural dimensions,” and “resource support.” This process not only organized the data but also initiated an exploration of its underlying structure.
(3) Employing a more integrative selective coding approach, we iteratively compared, synthesized, and refined these categories, ultimately distilling four overarching core themes from the complex data. These themes critically reveal the central issues and driving forces in the practice of student management and labor education in Chinese universities in the Xizang autonomous region (Table 3). These themes encompass students’ ambivalent attitudes toward management systems, the multifaceted educational functions of labor education, the evolving hybrid roles of teachers, and the structural tensions inherent in policy implementation. These four themes not only directly address the research questions posited in this study but also collectively delineate a complex landscape of higher education student management in Xizang, seeking creative equilibrium amidst multiple tensions within its unique cultural context. The entire analysis process emphasized inter-coder consistency, and through ongoing reflexive journaling, we documented the evolution of our understanding of the data, thereby ensuring the credibility and validity of our findings.

**Table 3:** Data analysis and thematic refinement.

Subject	Sub-subject	Core content	Phase
The ambivalent attitudes of students toward management systems	The voice of rational agreement	Understanding institutions as the “necessary framework” for maintaining order, ensuring fairness, and promoting harmony.	Student experience
	Emotional expression of disagreement	The frustration and sense of defeat stemming from the rigid and excessive institutionalization that constrains individual autonomy and growth.	Student experience
	The teacher’s insight and transformation	A paradigm shift in management philosophy from “controller” to “service provider” and “growth facilitator”, resolving tensions through democratic participation.	Teacher role
The multi-dimensional educational functions of labor education	Internalizing responsibility through practice	A paradigm shift in management philosophy from “controller” to “service provider” and “growth facilitator,” resolving tensions through democratic participation.	Student experience
	Constructing identity in cultural heritage	Labor education serves as a vital conduit, bridging modern pedagogy with cultural heritage, thereby solidifying cultural identity and regional belonging among the youth.	Cultural dimension
	A crucible of community consciousness	Collaborative labor emerges as a dynamic medium for fostering emotional connections among students from diverse ethnic backgrounds, reinforcing the consciousness of a shared Chinese national identity.	Cultural dimension
The transitional role transformation of teachers into composite professionals	Transitioning from “Authority” to “Partner”	The evolution of the mentor role toward that of an egalitarian, open “collaborative guide”, aimed at stimulating students’ academic autonomy and innovative potential.	Teacher role
	Building cultural bridges	Teachers act as cultural translators between “tradition” and modernity, facilitating a deeper “co-integration” among diverse cultural groups.	Teacher role/cultural dimension
Structural tensions in the implementation of management systems	Constraints of resource bottlenecks	The deficit in dedicated funding, practical platforms, and “dual-qualified” faculty leads to a diminishment of policy efficacy at the grassroots level.	Management system/resource support
	The urgency of localized adaptation	The inherent tension between uniform policies and local particularities necessitates creative adaptations in educational content and management approaches.	Management system
	Establishing a two-way feedback mechanism	Advocating for bottom-up communication channels to ensure that grassroots experiences substantively inform policy refinement and local contextualization.	Management system

### 4.3 Key findings

#### 4.3.1 Student perceptions of management systems: acceptance and resistance

Analysis and refinement of coded themes reveal a prevalent ambivalent attitude among students in China’s Xizang autonomous region regarding campus management systems. They generally acknowledge the necessity of these regulations while simultaneously yearning for greater flexibility. Management systems are perceived as an “essential framework” crucial for maintaining campus order, ensuring educational equity, and fostering social harmony within a diverse cultural landscape. However, the rigid, overly detailed, and, in some instances, “excessive” nature of their implementation leads students to feel a profound sense of constraint and helplessness, perceiving their personal autonomy and room for growth to be curtailed. This complex “love-hate” sentiment forms the fundamental undercurrent of student management in the Xizang autonomous region. Certainly, the student’s resistance to management, the tensions and failures encountered in the implementation of labor education, are not merely policy “implementation issues.” Rather, they are the inevitable outcomes of the interaction between individual agency and institutional governance within the multi-ethnic educational sphere, inherently interwoven into the processes of responsibility internalization and identity construction.

##### 4.3.1.1 Systems as foundational pillars of administration

A majority of students rationally recognize the foundational role of management systems. A law undergraduate student articulated this from a rule-of-law perspective: “*In my view, the school’s management regulations are akin to societal laws, establishing behavioral baselines for community members. Especially in a campus environment like ours, with multiple ethnicities and significant cultural differences, clear, uniform rules are particularly important. They minimize potential conflicts arising from cultural misunderstandings or differing customs and procedurally guarantee formal fairness for every student.”* (B03) This statement clearly reflects the students’ endorsement of the value systems in upholding basic order and procedural fairness within a cross-cultural campus setting. Another student from mainland China added, “*When I first arrived, I indeed found some regulations quite strict, such as the nightly dormitory checks. But after learning about some special circumstances, I gradually came to understand. In such a relatively unique region, the school bears significant security responsibilities, and these systems, in a sense, act as a ‘safety barrier’ for us.”* (B05) This understanding demonstrates the students’ capacity to look beyond immediate personal feelings and examine the functional role of management systems within a broader context. An undergraduate student shared a negative experience stemming from excessive bureaucracy: “*Once, we wanted to organize a campus environmental campaign, but it required going through multiple layers of approval. By the time the approvals came through, the event window had passed, and our classmates’ enthusiasm had been completely sapped. This kind of formalistic management not only wastes students’ time and passion but also makes us feel distrustful, even fostering a sense of alienation from the school.”* (B06) This negative experience reflects the potential detrimental effects that management systems can have when implemented.

##### 4.3.1.2 Treat us as adults

However, when management systems permeate numerous details of students’ learning and lives, especially when students perceive an underlying assumption of “distrust,” resistance naturally arises. One student, comparing the undergraduate and graduate stages, expressed evident frustration: “*During my undergraduate years, I could accept stricter university management, as I was just starting adulthood. But in graduate school, we need more autonomy in planning our research and flexibility in managing our academic time. Yet, many approval processes, like fieldwork sampling or equipment borrowing, remain incredibly cumbersome. It feels like we are presumed to be ‘individuals constantly needing supervision,’ and this atmosphere implicitly stifles the vitality of academic exploration.”* (Y05) This sentiment is quite common among graduate students, reflecting the tension between existing management systems and the developmental needs of high-level talent.

A senior student felt, “*Sometimes, it feels like the university lacks basic trust in us. For instance, a simple leave request requires layers of approval from the class advisor, counselor, and deputy secretary of the school. It’s as if university students cannot make basic judgments about whether they are truly sick or if there’s a genuine family emergency. This ‘managed’ feeling really dampens our motivation for self-management and our dignity as adults.”* (B07) These dissenting voices are not outright rejections of management but rather questions about its intensity and a deep yearning for respect and trust.

A teacher mentioned: “*Last year, we attempted to organize a highland ecological labor practice program in collaboration with mainland universities. However, due to a lack of effective communication with the local community, the program’s activities were disconnected from the production and lifestyle of local residents. This resulted in low participation rates, and ultimately, the project failed to achieve its intended outcomes. This reflects that, in implementing labor education, a lack of deep understanding of local realities can lead to project failure.”* (T02)

##### 4.3.1.3 Shifting the teacher’s role from manager to guide

The teaching staff, particularly counselors on the front lines of student affairs, possess a keen insight into this paradoxical sentiment. One educator pointed out that the key lies in a profound shift in management philosophy: “*We increasingly recognize that high-pressure, meticulously detailed ‘control’ can only govern students’ external behavior, not their inner selves. We are striving to promote a transformation, moving from the traditional ‘manager’ role to that of ‘service provider’ and ‘growth facilitator.’ For example, we are experimenting with implementing ‘democratic participatory management’ in classes, allowing students to collectively discuss and formulate certain class rules, and incorporating student hearing sessions for disciplinary matters. Encouragingly, the results have been excellent. The students’ sense of responsibility is continuously strengthening, shifting from a passive to an active stance, which is also the most significant effect of our management.”* (T05) This practical exploration points toward potential pathways for resolving tension through empowerment.

#### 4.3.2 The core role of labor education in cultivating responsibility and identity construction

In the Xizang Plateau, labor education in this multi-ethnic region offers profound implications beyond mere skills training. Through embodied experiences, it serves as a crucial arena for students to internalize a sense of responsibility, negotiate cultural identities, and strengthen regional belonging.

##### 4.3.2.1 Transforming slogans into inner convictions

The most direct function of labor education lies in its ability to translate abstract notions of responsibility and values into tangible, perceivable actions and experiences. One student shared a profound experience from participating in the campus “*Plateau Greenhouse” project: “Previously, in political thought classes, through slogans, I heard about ‘cherishing food’ and ‘loving labor,’ but they remained distant concepts. However, when you are truly at an altitude of over 3,000 meters, personally involved in loosening the soil, fertilizing, weeding, pest control, and finally harvesting vegetables, watching them grow under your care, the appreciation for the fruits of labor, the reverence for natural laws, and the sense of mission for the land beneath your feet arise spontaneously and are deeply etched into your being. This feeling is something that can never be learned by attending lectures in a classroom.”* (B02) This sense of responsibility, acquired through firsthand experience, is particularly profound and enduring due to the accompanying intense emotional engagement and cognitive restructuring. Another postgraduate student from the interior remarked after participating in technological services in a pastoral area: “*Giving injections to mice in a laboratory feels entirely different from going to the pastoral areas to help herders vaccinate yaks. The latter allows you to directly feel how your labor and knowledge are intrinsically linked to the livelihood of a family and the development of a community. This feeling of being needed has significantly enhanced my professional sense of responsibility.”* (Y02)

##### 4.3.2.2 Constructing identity through cultural inheritance: seeking “roots” and belonging

It is particularly noteworthy that when labor education is closely integrated with local cultural elements and traditional ways of life, it powerfully facilitates identity construction. This also represents a significant manifestation in the formation of identity. A Xizang student expressed deep emotion, her narrative filled with affective intensity: “*Although I am Xizang, I grew up in the city, and my living and educational environment were no different from those of children in the interior. My understanding of my ethnic culture and traditions, and ways of life, was superficial, and for a time, I felt my ethnic identity was blurred. It wasn’t until I participated in the ‘Xizang Traditional Handicraft Production’ labor course organized by the school, learning to weave ‘pulu’ and make pottery, that I felt I was touching the pulse of my ancestors, understanding the wisdom, esthetics, and lifestyle embedded within these artifacts. This was more than just a course; it allowed me to find the ‘roots’ of my culture, generating an unprecedented, genuine sense of pride and belonging in my ethnic identity.”* (Y01) This case vividly illustrates that labor education can serve as a bridge connecting modern education with cultural heritage, helping the younger generation to consolidate their cultural identity amidst modernization. On the other hand, for non-Xizang students, labor education plays a role in promoting cultural adaptation and regional integration. A student from the interior stated: “*By participating in the campus ‘Barley Planting Festival’ and ‘Wangguo Festival’ labor experiences, I am no longer just an observer or a tourist of the culture. I sweated and gained a visceral understanding of the production methods and reverence for nature that the people of this land have practiced for centuries, living ‘by the grace of heaven.’ This makes me feel less like a ‘guest’ here and more like someone genuinely participating and integrating, fostering a deeper emotional connection with my ‘second homeland.’*“(B08)

##### 4.3.2.3 Expert perspective: labor education as a crucible for community consciousness

Experts have affirmed the value of this integrated labor practice from a broader, more macroscopic viewpoint. An expert from the Xizang Plateau incisively remarked: “*In Xizang, labor education must not be isolated or a mere copy-paste from inland regions. When consciously integrated with local production and life, traditional culture, and ecological conservation, it transcends simple labor to become the most vivid, profound, and natural vehicle for ‘forging a strong sense of community among the Chinese nation.’ When students from different ethnic groups collaborate on common labor tasks, sweating toward a shared objective, the emotional connection and cultural resonance generated from this shared practice and experience are far more potent than any hollow indoctrination. It transforms community consciousness from a political imperative into a vibrant lived experience.”* (P02) This perspective profoundly reveals the unique socio-governance function of labor education in the Xizang region.

#### 4.3.3 Educators as cultural mediators and guides

Research in the unique context of China’s Xizang Autonomous Region reveals a significant deepening and expansion of the educator’s role. Beyond mere knowledge dissemination, educators function as “cultural mediators” and “collaborative guides,” adept at navigating cultural differences, providing individualized support, and bridging the gap between academia and society.

##### 4.3.3.1 A new paradigm of teacher-student collaboration: transitioning from authority to partnership

This evolution is particularly pronounced in postgraduate education, where the instructor’s role is shifting from a traditional, hierarchical “authoritative” stance to a more egalitarian, open “partner” or “guiding” model. One student’s description of their relationship with their advisor is illustrative: “*My advisor never directly tells me the answers or dictates what I must do. Their typical questioning involves, ‘What are your thoughts?’, ‘What literature have you consulted?’, ‘How do you plan to design the experiment to tackle this technical challenge?’ They provide me with crucial laboratory resources, academic direction, and broad research trajectories, but profoundly respect my individual research interests and specific ideas. We frequently discuss, even debate, which feels more like a team striving for a common research goal, with them as a senior partner and coach.”* (Y03) This collaborative dynamic significantly fosters students’ academic autonomy, sense of ownership, and innovative potential. An educator candidly admitted, “*Times have changed. Especially with today’s easy access to information, an educator’s authority is no longer built on knowledge monopoly but on their ability to ignite students’ intrinsic motivation and offer critical guidance during moments of uncertainty.”* (T04)

##### 4.3.3.2 Builders of cultural bridges: navigating tradition and modernity

Educators, especially those from ethnic minority backgrounds or long-term residents deeply familiar with local Xizang culture, implicitly fulfill an indispensable role as “cultural bridges.” An experienced educator who has worked in Xizang for 15 years shared their unique perspective: “*Much of my work involves ‘translation’—not just linguistic, but also cultural and psychological. I need to explain to colleagues and students from other regions why a particular Xizang festival is important and its underlying cultural significance; how to interpret certain phrases or behaviors within the local cultural context to avoid unintentional offense or misunderstanding. Concurrently, I assist Xizang students in better understanding and adapting to the general rules and academic norms centered around the modern university system, encouraging them to embrace new knowledge with greater openness and confidence, rather than remaining insular. This process, at a micro-level, promotes ‘integration’ among different cultural groups, rather than mere formal ‘coexistence’“.* (T07) This function is crucial for cultivating an inclusive and understanding campus cultural atmosphere.

Another educator elaborated on the supportive role of teachers from a different angle: “*Here, we recognize that educators must not only teach effectively but also serve as ‘life mentors’ and ‘emotional supporters’ for our students. The students here are generally sincere and kind-hearted, but due to factors such as region, culture, and family background, they are more prone to experiencing psychological confusion and emotional fluctuations. We often leverage relaxed, informal settings created by practical activities like labor education to engage in deeper conversations, understand their challenges beyond academics, and provide them with comprehensive humanistic care and support that extends beyond scholarly pursuits.”* (T06) This indicates that in the Xizang Autonomous Region of China, the educator’s function of “care” is imbued with higher expectations and greater significance.

#### 4.3.4 Institutional gaps and local adaptation

At the national level, educational policies frequently encounter a “last mile” challenge during their implementation in Xizang higher education institutions. A discernible gap exists between the envisioned policy blueprint and the intricate local realities, stemming from resource constraints and insufficient localization efforts.

##### 4.3.4.1 Resource bottlenecks

The primary constraint on the effective implementation of policies is a scarcity of resources, particularly human capital. As one expert candidly stated, “*From higher-level directives, the requirements for class hours, content, format, and objectives for labor education are very clear, even mandatory. We fully support this as it’s essential for education. However, at the execution level, the greatest and most realistic challenge is ‘insufficient resources.’ Firstly, dedicated funding is limited and its utilization process is rigid. Secondly, there’s a lack of stable, diverse, and high-quality off-campus practical bases. Most critically, we have a severe shortage of ‘dual-qualified’ labor education teachers who understand modern educational concepts, are familiar with local industrial characteristics, and possess the ability to guide student practice. Most current teachers are either ‘transferred’ from other disciplines or ‘part-time,’ significantly compromising their professionalism.”* (P05) This predicament of having the will but lacking the means is a pervasive issue faced by many frontier universities.

##### 4.3.4.2 Urgency of localization

A significant contradiction arises from the “one-size-fits-all” approach of policies or their insufficient consideration of local specificities. A frontline teacher provided a vivid example: “*For instance, national-level labor education syllabi or guidelines might recommend certain industrial assembly line internships or modern agricultural projects suitable for plain regions. These might be excellent for factories or farms in eastern China, but utterly unfeasible in high-altitude, cold environments and vastly different from students’ lived experiences. If we do not proactively and creatively ‘localize’ these, transforming them into projects related to highland ecological protection, Xizang traditional handicrafts, characteristic cultural tourism, or clean energy development, students will perceive this labor as ‘someone else’s labor,’ an imposed formality irrelevant to them, significantly diminishing their engagement and the intrinsic educational effects.”* (T01) This discourse profoundly reveals that educational content lacking cultural and regional appropriateness struggles to ignite students’ intrinsic motivation and emotional resonance.

A cadre responsible for student management added from a student governance perspective: “*Student management systems are similar. For example, dormitory management, if completely replicating the highly standardized models from inland regions, might inadvertently overlook or suppress the living habits and cultural expressions of some ethnic minority students. We need to implement some humane and flexible ‘micro-adjustments’ while adhering to fundamental principles like campus safety and public health. This requires management acumen and, more importantly, cultural sensitivity.”* (T03)

##### 4.3.4.3 Grassroots demand for a two-way feedback mechanism

Consequently, grassroots educators strongly advocate for the establishment of an effective, institutionalized two-way feedback mechanism. One leader summarized: “Policy formulation and decision-making should not be a one-way, closed-loop issuance of directives. There must be a smooth and valued channel through which our grassroots voices—our unique challenges, our explored effective practices, our suggestions for revisions based on local realities—can be heard promptly and accurately by decision-makers, and can substantially influence policy revision and optimization. Only then can macro-level policies achieve more refined ‘local adaptation’ and truly take root, grow, and flourish on the land of the plateau frontiers, rather than remaining suspended in the air.” (P04) This call points to the crucial issue of “top-down and bottom-up linkage” in the modernization of the education governance system.

#### 4.3.5 Comparative analysis of differences across groups

To systematically compare differences across groups, we employed a “group-theme” matrix analysis. Specifically, we first identified four core themes and then compared each theme’s manifestation across different groups. For instance, under the theme “Students’ Ambivalent Attitudes Toward Management Systems,” undergraduates focused more on the rigid enforcement of systems, graduate students emphasized the tension between systems and academic autonomy, while experts analyzed the rationality of system design from a policy perspective. Within the theme “Multidimensional Educational Functions of Labor Education,” Tibetan students emphasized cultural identity recognition, while non-Tibetan students prioritized cultural adjustment and regional integration. Regarding the theme “Transformation of Teachers’ Composite Roles,” young faculty highlighted the “growth facilitator” role, whereas senior teachers stressed the “cultural bridge” role. This systematic comparison reveals the applicability and variations of themes across groups, enriching the depth of research findings.

### 4.4 Major challenges and contributing factors in labor education at Xizang higher education institutions

#### 4.4.1 Structural challenges: misalignment between policy support and resource implementation

This predicament is primarily characterized by a significant disconnect between ambitious top-level policy design and the paucity of foundational support. Despite the central government’s unprecedented strategic emphasis and promotion of labor education, crucial supporting resources such as dedicated funding, the cultivation and recruitment of specialized faculty, the development of sustainable and high-quality practical platforms, and a scientific, process-oriented evaluation system have not been allocated synchronously, adequately, or precisely to Xizang, a frontier region. The underlying reasons stem from the historically short development period and relatively weak foundation of higher education in border areas, which inherently limit their capacity for resource acquisition, absorption, and transformation. When rapidly responding to unified national policy requirements, universities and local education administrative departments at the meso-level encounter evident efficiency bottlenecks and capability deficits in resource integration, project planning, and channel development, leading to diminished effectiveness during policy transmission.

#### 4.4.2 Adaptive challenges: tension between unified requirements and local characteristics

This refers to the inherent conflict between the universal and unified demands of national policies and the unique, differentiated realities of Xizang. Uniform national policy guidelines, curriculum standards, and evaluation systems are difficult to fully align with Xizang’s distinctive high-altitude geographical environment, its multifaceted cultural traditions, its varied developmental stages and industrial demands, and the specific psychocultural characteristics of its students. When labor education programs lack necessary and sufficient “localization” through creative adaptation, they risk becoming maladaptive, failing to deeply connect with students’ lived experiences, interests, and future development. Consequently, this hinders the cultivation of sustained intrinsic motivation and profound emotional resonance, ultimately weakening labor education’s potential efficacy in constructing identity, fostering cultural integration, and enhancing social cohesion.

The fundamental causes can be attributed to the inherent structural tension between the universality and standardization logic of macro-level policies and the specific, diverse needs of frontier regions. Currently, the adaptive and innovative capacities of meso-level organizations (universities), as well as the understanding and execution capabilities of micro-level individuals (teachers and students), are insufficient to fully and effectively bridge this macro–micro gap. As one expert noted, “*From the perspective of higher-level documents, very clear, even stringent requirements are set for the hours, content, format, and objectives of labor education. However, at the implementation level, the biggest and most realistic difficulty, frankly, is the lack of resources. We are extremely short of dual-qualified labor education teachers who understand modern educational concepts, are familiar with local industrial characteristics, and possess the ability to guide student practical activities.”* (P03). Another expert emphasized, “*If we do not undertake proactive, creative localization adaptations, students will perceive this labor as someone else’s labor, an imposed formalism unrelated to them, greatly diminishing their participation and the intrinsic educational effect.”* (P06). A manager appealed, “*Policy formulation and decision-making processes cannot be unidirectional, closed-off directive issuances. There must be a smooth, valued channel through which our grassroots voices… can be heard promptly and accurately by decision-makers and can substantively influence policy revisions and optimizations.”* (P07).

### 4.5 Psychological evolution and mechanistic interpretation of labor education

This study reveals the deep-seated pathways of labor education’s impact from a psychological mechanism perspective, which can be summarized as a progressive psychological chain: “labor education - emotional resonance - internalization of responsibility - formation of cultural identity.” First, labor education sparks emotional resonance through embodied participation. Students generate intense emotional experiences during labor, transforming abstract labor values into concrete feelings. Second, this emotional resonance promotes the internalization of responsibility. Students transition from being required to labor to proactively assuming responsibility, shifting their values from external normative constraints to internal psychological drivers. Finally, this internalized sense of responsibility extends into cultural identity. When labor practices are integrated with local culture and traditional festivals, students develop cross-cultural emotional connections and a sense of belonging through collective labor. This psychological chain reveals that labor education is not merely a teaching activity but a profound process of psychosocialization. It offers a new theoretical perspective for understanding the socialization and identity construction of students in multi-ethnic universities within the context of cultural diversity.

## 5 Discussion

### 5.1 Distinguishing this study from existing research

The findings of this study resonate with, and importantly, diverge from and advance upon, existing research both domestically and internationally. These distinctions primarily stem from the unique theoretical perspectives, methodologies, and research subjects employed herein. Firstly, this study corroborates several propositions advanced by prior scholarship. The foundational role of labor education in cultivating students’ practical skills and sense of responsibility aligns with domestic consensus (Corson, 1985). As an effective experiential learning modality, it resonates with the core tenets of teaching, learning, and doing emphasized in international “service-learning” and “experiential learning” frameworks (Blackburn, 2005). Furthermore, our observation of students’ resistance to managerial rigidity and their yearning for autonomy echoes the universal demand for personalized development in higher education globally (Raaper, 2021). This study meticulously delineates empirical findings, interpretive extrapolations, and policy-oriented arguments, calibrating each class of assertion appropriately. Empirical conclusions are rigorously substantiated by data and facts. Interpretive extrapolations are clearly demarcated as inferential. Policy-oriented arguments, in turn, propose actionable recommendations predicated on the research outcomes. While certain interpretive extrapolations retain an exploratory character, the explicit categorization of assertion types effectively ensures the rigor and normative compliance of the argumentation.

However, within the unique context of China’s Xizang, border, and multi-ethnic plateau regions, this study unveils deeper insights that transcend current research paradigms. The divergence lies in several key areas. International “service-learning” and “experiential learning,” often rooted in Western individualistic cultures, place a particular emphasis on the role of the instructor (Chang, 2020). Through embodied communal labor and cultural integration practices, it serves as a micro-governance mechanism that fosters the construction of national identity and promotes social cohesion. Concurrently, compared to the majority of domestic research that predominantly focuses on policy interpretation or activity descriptions, our study, employing a bottom-up qualitative approach, vividly portrays students’ ambivalent attitudes toward management systems and the multifaceted role of educators as cultural mediators navigating the tensions between macro-level policies and micro-level practices. These dynamic, embodied interaction processes have not been adequately captured by existing macro-level studies.

### 5.2 Reasons for divergence from existing research

The foundational divergence of this study stems from its integrated theoretical framework, which synergizes theories of practice education, identity, and educational equity with rigorous qualitative research methodologies. This approach allows us to move beyond generalized policy statements and acutely discern the specific governance logics and deep cultural interaction mechanisms inherent in educational practices within unique geographical, cultural, and political contexts.

#### 5.2.1 The functional deepening of labor education: from skill training to governance mechanisms

International scholarship often confines “labor education” to vocational skill training or livelihood education (Koneberg et al., 2025), while domestic discourse predominantly focuses on its value in “cultivating virtue and fostering talent” and developing practical abilities (Li, 2023). This research, however, reveals that in universities situated in the Xizang Plateau, border regions, and multi-ethnic areas, the function of labor education extends far beyond these confines. Through embodied practice, cultural integration, and community building, it evolves into a micro-level governance mechanism for multicultural education. While exhibiting formal similarities to “service learning” (Block and Bartkus, 2019) and “experiential learning” (Seaman et al., 2017) within international discourse, its core objective transcends the Western individualistic emphasis on personal critical consciousness and social skills. Instead, it prioritizes the cultivation of national identity, the promotion of cultural integration, and social cohesion within the distinctive milieu of the Xizang Plateau, border regions, and multi-ethnic areas. This finding addresses the limitations of international theories in explaining educational practices in non-Western, multi-ethnic contexts and elevates the domestic understanding of labor education from a descriptive level of “activity” (Zhang, 2022) to a profound dialog with core theories of identity and educational equity.

#### 5.2.2 Regulations of student management versus student and faculty realities

Existing research on university management in the Xizang Plateau, border regions, and multi-ethnic areas predominantly adopts a top-down perspective, excelling in policy articulation and institutional analysis but falling short in illustrating the complex interactions during policy implementation at the grassroots level. By amplifying the “voices” of students and faculty, this study vividly portrays the central tension between regulation and autonomy in student management. The students’ ambivalent mindset—understanding the necessity of regulations yet yearning for flexibility—and the faculty’s attempts to transition from “managers” to “facilitators” represent nuanced micro-level experiences largely uncaptured by current macro-level research. This “bottom-up” approach reveals that management is not a mere execution of policy but a dynamic process replete with negotiation, adaptation, and innovation, thereby substantially enriching our understanding of the complexities of university management in the Xizang Plateau, border regions, and multi-ethnic areas.

#### 5.2.3 Redefining the teacher’s role from knowledge authority to cultural mediator

Existing research on the roles of university faculty primarily focuses on “teaching and nurturing students” and “mentoring guidance” (Yang, 2023). This study reveals a profound and multifaceted transformation of the teacher’s role within the unique context of universities in China’s Xizang, border, and multi-ethnic plateau regions. Teachers are not merely purveyors of knowledge and academic guides, but, crucially, cultural mediators. They engage in “translation” and mediation between tradition and modernity, and between the nation and local communities, striving to foster a transition from formal “inclusion” to substantive “co-integration” among diverse cultural groups. This role redefinition resonates with international theories of “intercultural management” and the concept of “communities of practice.” However, it uniquely emphasizes the teacher’s identity as an active governance collaborator within the specific politico-cultural backdrop of the Xizang Plateau, border, and multi-ethnic regions, thereby significantly augmenting existing theories of teacher roles. Our research expands the psychological understanding of how experiential education promotes identity integration and civic consciousness formation in multicultural settings. By situating labor education within the psychosocial dynamics of internalized responsibility and cultural adaptation, this study achieves a deep integration of educational practice and psychological theory, offering new insights for global research on experiential and intercultural education.

### 5.3 Limitations and future research

Despite our diligent efforts to conduct a thorough and rigorous investigation, this study acknowledges several limitations. (1) Sample Representativeness and Generalizability Limitations. Our study employed purposive sampling, prioritizing the richness and depth of information over statistical representativeness. While all participants were recruited from universities in Xizang Plateau, border regions, and multi-ethnic areas, ensuring a diverse sample, our findings may not fully capture the situation across all higher education institutions in these regions, nor can they be directly generalized to other ethnic minority regions in China’s frontiers. Variations in resources, history, and traditions among different institutions could influence the specific models of student management and labor education practices. (2)Timeliness and Subjectivity of Interview Data. The data was collected at a specific point in time, reflecting perceptions and experiences within a particular policy cycle and practice phase. Educational policies and practices are dynamic; thus, the timeliness of our conclusions warrants future validation. Furthermore, interview data relies on participants’ self-reports, inherently carrying subjectivity and recall bias. Although we employed triangulation (comparing perspectives from different groups) to enhance objectivity, complete avoidance of these issues was not possible. (3) Potential Cultural Understanding Gaps. While the research team strived for cultural sensitivity and included members familiar with local cultures, as external researchers, our understanding of the deeper cultural nuances in Xizang Plateau, border regions, and multi-ethnic areas may be incomplete. This cultural distance could impact the precise interpretation of the cultural logic underlying certain interview content. (4) Lack of Longitudinal Tracking and Quantitative Supplementation. As a cross-sectional qualitative study, this research illuminated phenomena, mechanisms, and tensions, but could not establish causality between labor education and factors like student identity or sense of responsibility, nor could it depict the dynamic trajectories of these factors over time. The absence of quantitative data to support policy effects or resource allocation meant that discussions on “resource bottlenecks,” for instance, remained at a qualitative descriptive level.

Future research directions aim to deepen and expand upon our findings: (1) Conduct Longitudinal Tracking Studies. We plan to conduct follow-up interviews over 2–3 years with a subset of students from universities in Xizang, border regions, and multi-ethnic plateau areas. This will allow us to explore shifts in their perceptions of management systems across different stages of their academic careers and investigate the long-term impact of labor education on their identity formation and career choices, thereby revealing the dynamic efficacy of educational practices. (2) Undertake Cross-Regional Comparative Research. We intend to conduct similar research in universities within other ethnic regions, such as Qinghai, Xinjiang, and Inner Mongolia. Through comparative analysis, we aim to discern the uniqueness of the experiences on the Xizang Plateau, border regions, and multi-ethnic plateau areas, as well as identify common patterns in university governance across ethnic regions. This will enhance the universality and explanatory power of our theoretical framework. (3) Employ Mixed-Methods Research. In subsequent studies, we will design large-scale questionnaire surveys to quantitatively measure core variables identified in our findings (e.g., management satisfaction, perceived effectiveness of labor education, strength of Chinese national identity). This quantitative data will complement and validate our qualitative findings, constructing a more comprehensive and persuasive evidence chain. (4) Deepen Action Research and Collaboration. We aspire to establish more profound collaborative relationships with universities in Xizang Plateau, border regions, and multi-ethnic plateau areas. Transitioning from being mere “researchers” to “collaborative actors,” we aim to jointly design and evaluate labor education programs or management optimization strategies that are more locally appropriate, based on our research findings. This will directly channel academic research into improving local educational practices.

## 6 Conclusion

### 6.1 Key findings

This study reveals that student management in universities within Xizang, border, and multi-ethnic plateau regions of China is a complex process that seeks creative balance through the triple interaction of macro-level policies, meso-level organizations, and micro-level individuals. (1) Students exhibit a pervasive “ambivalent mindset” toward management systems. While they rationally acknowledge the system’s necessity as a “safeguard” for maintaining campus order and ensuring cross-cultural fairness, they emotionally and practically feel helpless and resistant due to the limitations placed on their personal autonomy by rigid and excessive enforcement, calling for a more flexible and trust-based management philosophy. (2) Labor education demonstrates “multi-dimensional educational functions” beyond mere skills training. Through embodied experiences, it serves as a catalyst for students to internalize a sense of responsibility. When integrated with local cultural traditions, it becomes a crucial arena for the younger generation to construct cultural identities and strengthen regional belonging, ultimately sublimating into a vibrant crucible for forging a strong sense of community among the Chinese nation. (3) The role of teachers is undergoing a profound “composite transformation.” They are evolving from traditional authorities on knowledge and managers into equal academic partners and growth mentors. Particularly in multicultural settings, they function as indispensable “cultural intermediaries,” building bridges of communication between “tradition” and “modernity” to promote deeper cultural integration. (4) The implementation of national policies universally encounters “structural tensions.” This is most prominently reflected in the misalignment between “top-level design” and “grassroots resource provision,” as well as the incompatibility between “unified requirements” and “local characteristics.” There is a strong call from the grassroots for the establishment of effective two-way feedback mechanisms to achieve refined local adaptation of policies.

### 6.2 Theoretical contributions

#### 6.2.1 Innovation in theoretical perspective

An analytical framework of “labor education as a nexus for multicultural governance” has been constructed. This study theorizes the regional labor education practices in Xizang, border, and multi-ethnic plateau regions of China as a governance mechanism connecting national integration, cultural inheritance, and individual development. This perspective transcends the traditional view of labor education as a mere pedagogical tool, positioning it within a broader context of social governance and identity politics, thereby offering a new paradigm for understanding the educational functions in non-Western, multi-ethnic states.

#### 6.2.2 Deepening of practical pathways

The micro-level mechanisms for realizing “inclusion to integration” have been elucidated. Through meticulous empirical research, this study indicates that achieving cultural integration in the Xizang Plateau, border, and multi-ethnic regions is not solely reliant on ideological advocacy or policy mandates but depends on a series of micro-level practices. These mechanisms translate the macro-level political objective of “community consciousness” into perceptible and participatory life experiences, providing actionable pathways for deeper social integration.

#### 6.2.3 Methodological exemplification

A “bottom-up” research path for cross-cultural education has been established. This study has established a bottom-up approach to cross-cultural education research. Through in-depth qualitative interviews, it successfully captured the vivid experiences of individuals within policy and institutional frameworks. This demonstrates that in research concerning ethnic regions and sensitive topics—such as the Xizang Plateau, border areas, and multi-ethnic regions—the authentic voices of interviewees provide valuable methodological insights for subsequent related studies.

#### 6.2.4 Establishing research contributions

First, empirical findings are based on direct observations from interview data, such as students’ ambivalent attitudes toward management systems and the multidimensional educational functions of labor education. Second, interpretive extensions derive theoretical inferences from empirical discoveries, such as the psychological chain “from action to identification.” Third, policy-oriented arguments propose recommendations based on research findings, such as establishing a two-way feedback mechanism. We emphasize that the theoretical contributions of this study primarily stem from empirical findings and interpretive extensions, while policy recommendations represent a natural extension of these theoretical insights.

### 6.3 Practical recommendations

(1) University administrators should adopt a transformative management philosophy, fostering a governance structure built on trust. This involves a paradigm shift from “control and supervision” to “guidance and trust,” granting students greater autonomy in both graduate and undergraduate management. Establishing a “democratic participatory” management mechanism will empower students with a sense of ownership and self-governance. Furthermore, the localization and curricular integration of labor education are crucial. Developing labor education programs and curricula that incorporate regional distinctiveness—focusing on plateau ecology, Xizang culture, and characteristic industries—will ensure that labor education is both practically grounded and intellectually enriching. The cultivation of a “dual-qualified” labor education faculty, supported by dedicated funding, will foster educators possessing both professional expertise and cultural acumen, thereby providing sustained, professional talent support for labor education initiatives.
(2) Policymakers must enhance policy flexibility to promote regional adaptability. Drawing upon global best practices and contextualizing them to local realities, guidance documents should provide ample space for localized exploration in Xizang Plateau, border, and multi-ethnic regions, allowing for tailored, differentiated approaches. The establishment of a bidirectional policy feedback mechanism, integrating both top-down directives and bottom-up insights, will create a dynamic feedback loop. This system will systematically incorporate practical challenges and innovative experiences from the university frontline, informing policy optimization. Enhancing the precision and sustainability of resource allocation is paramount. Addressing the practical difficulties faced by universities in plateau and border regions requires targeted, stable, and flexible financial and human resource support, mitigating the risk of intentions exceeding capabilities.
(3) University faculty members should strengthen cultural sensitivity and intercultural guidance capabilities. Proactively updating pedagogical perspectives and enhancing skills in intercultural communication and project-based learning design will position educators as guides for students’ academic and cultural development. Leveraging the informal settings of labor education for enhanced student development is essential. Effectively utilizing informal pedagogical arenas, such as labor education activities and social practice, will facilitate understanding of student needs through relaxed interaction, offering empathetic psychological care and emotional support.

## Data Availability

The original contributions presented in the study are included in the article/Supplementary material, further inquiries can be directed to the corresponding author.
