# Supplementary material for: The voice of the plateau: a qualitative interview study of student management and educational practices in higher education in Xizang Autonomous Region
Source: Front Psychol. 2026 Mar 4;17:1754922. doi: 10.3389/fpsyg.2026.1754922 (PMC12996261; doi:10.3389/fpsyg.2026.1754922)
Supplement: Supplementary file 1 [file Supplementary_file_1.docx]

**Appendix**

**Interview Outline**

1. Undergraduate Interview Outline:

1. How do you currently understand and perceive university student management systems?

2. What are your participatory experiences in labor education, disciplinary requirements, and group activities? Are there any particularly memorable or positive experiences?

3. What impact do you believe school management has on students' academic performance, daily life, and psychological well-being? What measures do you find most engaging?

4. How does your ethnic cultural background influence your campus life and experiences with student management?

5. Discuss the changes in your understanding of responsibility, discipline, and autonomy during your time at university.

II. Graduate Student Interview Outline:

1. Compared to your undergraduate studies, what differences have you experienced in student management during your graduate program?

2. What do you perceive as the relationship between academic research, research tasks, and university management?

3. How do you understand the attitude toward and significance of labor education or social practice?

4. What role do you believe supervisors play in management and guidance (i.e., the mentor-mentee relationship)?

5. What are your views on balancing autonomy and discipline within higher education practices?

III. Faculty Interview Outline:

1. In student management, what do you consider to be the teacher's role? What approaches are most effective for your students to accept?

2. In the current phase, how do you understand and implement labor education and student practice activities?

3. How do you understand the attitude toward and significance of labor education or social practice?

4. Working in the Tibet region, what cultural differences and management challenges have you encountered, and how have you addressed them?

5. What are your views on educational policies in Tibet, and how effectively are they being implemented?

6. In student management, how do you enhance students' sense of belonging and participation? How do you reduce student resistance?

IV. Expert Interview Outline:

1. How do you view the overall characteristics and specificities of student management in Tibetan universities?

2. What value and unique role do you believe labor education holds in universities in the Tibet region?

3. How do you understand the attitude toward and significance of labor education and social practice?

4. What significant meaning do you attribute to Tibetan university student management in terms of educational equity, ethnic unity, and social responsibility?

5. We observe a gap between policy execution and practice; in which areas do you believe improvements are needed?

6. What are the future trends you foresee for student management and educational practice in Tibetan universities, and what recommendations do you have?
